# Chronic Colitis: Its Causation, Diagnosis and Treatment

**Published:** 1914-09

**Authors:** 


**Chronic Colitis : its Causation, Diagnosis and Treatment.** By George Herschell, M.D., and Adolphe Abrahams, M.D. Pp. 273. London : Longmans, Green & Co. 1914. 6s. net.

Although this book is entitled *Chronic Colitis,* the authors expressly exclude ulcerative colitis, " except in so far as it relates definitely as a complication to chronic colitis." They make a clinical classification into chronic colitis (*a*) with, and (*b*) without, excessive secretion of mucus, and (*c*) muco-membranous colitis. Clinically there is no doubt, whatever its pathology may ultimately be shown to be, that the latter disease gives a distinct symptom-grouping. The difficulties of such a clinical classification appear in the second chapter on pathology, where a classification founded upon a distinctly anatomical basis is given, and in this and the following chapter on causation conditions are included which lead on to ulcerative colitis. This is apt to produce confusion in the reader's mind, as in discussing the disease the authors do not clearly distinguish between the two conditions. We think that in a work on colitis the different forms of ulcerative colitis should be included, because from their intractable nature, their tendency to progressive involvement of fresh areas of the bowel, and their often fatal termination, they are certainly the most important.

The scope of the work is therefore with what we may term the minor forms of colitis, no doubt a sufficiently troublesome group of diseases, and often misapprehended. In these forms of colitis the authors quite rightly lay stress on the paramount importance of previous constipation in the causation, and also rightly combat the erroneous idea, still widely prevalent, that diarrhœa of some kind is a constant symptom of these disorders, whereas many cases are attended throughout by constipation.

We do not regard the classification into clinical types of the disease given in this book as of value. The first four depend merely upon individual variations in the clinical course of the disease, and may probably all be referred to the effects of long-continued constipation, and we see no advantage in labelling other forms pseudo-malarial, pseudo-typhoid, etc., but rather the reverse. Malaria is a term which has now a definite signification, to which it should be restricted.

As to the chapter on diagnosis, though the importance of the sigmoidoscope in examination is recognised, we think that this section in a book professedly dealing with affections in which the sigmoidoscope is especially valuable, should be much fuller, especially as to morbid appearances ; again, in dealing with the question of occult blood in the fæces, no advice is given as to the most useful tests ; again, in the section on dissolved albumin in the stools, the diagnostic indications are not given precisely enough. A few additions of a similar kind and a little further elaboration would greatly increase the value of the section on diagnosis. The larger part of the book is taken up with the treatment, which is therefore very fully discussed. The practitioner will find in it a number of useful hints, and there is a section on the cooking and preparation of foods which will be found very helpful. On the whole, allowing for individual differences of opinion as to the value of particular remedies, the advice given is sound and practical. This section is perhaps rather discursive in parts, and the number of remedies given may be somewhat bewildering to one who desires direct help in the treatment of a special case ; a short summary of the measures the authors consider best would add to its practical utility. We have no doubt that our readers will find this book a help in the treatment of a troublesome class of cases. There is a good index.

